# Integrating Sensor Models in Deep Learning Boosts Performance: Application to Monocular Depth Estimation in Warehouse Automation

**DOI:** 10.3390/s21041437

**Published:** 2021-02-19

**Authors:** Ryota Yoneyama, Angel J. Duran, Angel P. del Pobil

**Affiliations:** 1 Department of Computer Science, Jaume I University, 12071 Castellon, Spain; al388242@uji.es (R.Y.); pobil@uji.es (A.P.d.P.); 2 Department of Interaction Science, Sungkyunkwan University, Seoul 110-745, Korea

**Keywords:** deep learning in sensing, robot sensors, vision/camera based sensors, 3D sensing, monocular depth estimation, warehouse automation, optic flow

## Abstract

Deep learning is the mainstream paradigm in computer vision and machine learning, but performance is usually not as good as expected when used for applications in robot vision. The problem is that robot sensing is inherently active, and often, relevant data is scarce for many application domains. This calls for novel deep learning approaches that can offer a good performance at a lower data consumption cost. We address here monocular depth estimation in warehouse automation with new methods and three different deep architectures. Our results suggest that the incorporation of sensor models and prior knowledge relative to robotic active vision, can consistently improve the results and learning performance from fewer than usual training samples, as compared to standard data-driven deep learning.

## 1. Introduction

In the last years, deep learning has become the mainstream paradigm in computer vision and machine learning [1]. Following this trend, more and more approaches using deep learning have been proposed to address different problems in sensing for robotics. However, robots pose a number of challenges for this methodology, that relate to the fact that robot sensing is inherently active [2]. This active nature also offers opportunities that have been exploited for years in the context of active vision [3]; for instance, more information can be extracted from sensory signals by incorporating knowledge about the regular relationship between them and concurrent motor actions [4]. Similarly, spatial and temporal coherence resulting from embodiment can be exploited, for example by taking advantage of the correlation of consecutive images or those taken from slightly different viewpoints [2]. In contrast, data-intensive computer vision relies primarily on enormous amounts of decontextualized images.

As it is well known, the performance of a neural network is directly related to the adequacy of the training set, and for deep learning, a huge amount of data is typically a must. Whereas these datasets are normally available for purely data-driven computer vision or machine learning approaches, performance is usually not as good as expected when they are directly used for applications in robot vision or, in general, for problems in robot learning for which results are brought about by motor actions in robotics applications [2]. In these applications, relevant data is often scarce and, moreover, those exhaustive datasets would turn out to be unfeasible. This calls for novel deep learning approaches that can offer a good performance from fewer than the usual number of training samples.

**Figure 1:**
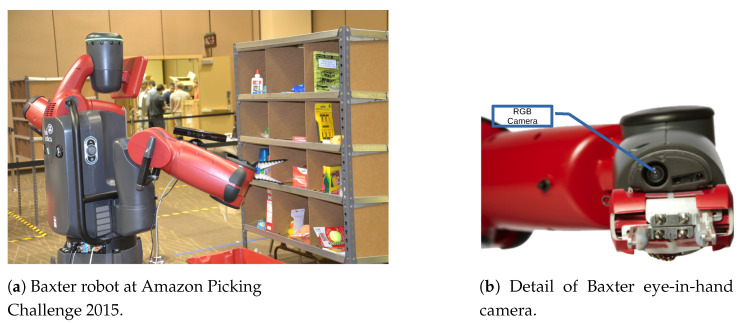
(**a**) Baxter robot with UJI RobInLab team at the Amazon Picking Challenge 2015. Manipulating items within the confined space of the shelf poses a number of challenges in terms of visibility and maneuverability. This could not be accomplished with the RGB-D sensor shown in the image that was mounted on the robot’s elbow. (**b**) shows a detail of Baxter’s fully integrated built-in eye-in-hand visual sensor that we propose to use for 3D depth estimation as a complement to the RGB-D sensor.

In this paper, we address the above issues by means of new methods and the incorporation of models and prior knowledge based on active vision. The specific robotic problem we solve here is monocular depth estimation in an eye-in-hand configuration. The main goal here is to pick and place objects for which the depth cue is the necessary starting point for most grasping algorithms [5,6,7]. This problem arises in applications such as warehouse automation for which adequate datasets are nonexistent (for instance the most popular datasets for depth estimation such as the KITTI dataset [8] or the Pandora dataset [9] are oriented to depth estimation by vehicles). Additionally, manipulation in a confined space would require an RGB-D sensor in hand and, even though state-of-the-art RGB-D cameras are more compact, they are not comparable with the Baxter fully integrated built-in eye-in-hand cameras (see Figure 1a,b). In addition, RGB-D sensors have problems with reflective and transparent objects [10,11]. Our goal is, then, not so much to replace existing 3D sensors but to complement them in situations where there may exist some difficulty in using them. The proposed algorithms allow—using deep learning techniques—to estimate the depth in a static scene from small displacements of a more compact sensor, such as an RGB camera.

Model-based approaches are usually opposed to purely data-driven methods—such as deep learning—but the use of environment models in combination with model-free algorithms is a promising trend towards more efficient reinforcement learning [12]. In a similar way, we explore this research avenue by integrating models and prior knowledge pertaining to our previous work regarding the relationship between the optical flow and the displacement of the camera [13]. With this aim, we incorporate into the generic data-driven deep learning techniques the knowledge of the specific parameters of our sensor. More specifically, we propose here three deep network architectures in such a way that information from a modelled and parameterized sensor is considered sequentially in their design, namely the estimation of image displacements based on the camera model; the optical flow estimation based on the correlation of two consecutive images and the subsequent correlation with the change in camera position; the estimation of the camera displacement from a depth image. The incorporation of each model consistently improves the results and learning performance with a considerably smaller data consumption cost of training, as compared to pure data-driven deep learning.

### Related Work

In the case of deep learning for object recognition, some works have taken advantage of active vision [14], and even a dataset has been recently proposed that somehow includes temporal consistency [15]. For an up-to-date compilation of the literature on deep learning in robotics and interactive perception, see [2,4], respectively.

Inferring a depth map using a monocular camera or a single eye is relatively easy for humans, but it is difficult for computational models. In computer vision, a number of methods and algorithms have been established for estimating the depth of a scene using a single camera or image. For instance, by applying patches to determine the pose of planes in a single image, it is possible to generate the depth map with a single image [16]. Additionally, from a stream of images, the depth map can be deduced if the velocity of the camera is known [17]. Recent results on structure from motion with a monocular camera are based on feature tracking and triangulation methods [18]. Biology is a source of inspiration in this field too—Antonelli et al. [13] replicated fixational head and eye movements in primates together with the resulting optical flow to estimate depth.

Related work on monocular depth estimation with convolutional neural networks (CNN) can be categorised according to the number of input images (single or multiple) and the learning approach (supervised or unsupervised). A multi-scaled deep network for supervised learning was proposed to infer the depth map from a single image [19]. Others followed this single-image approach by considering computational random fields [20] or using long short-term memory and recurrent neural networks [21]. Even though it is possible to reconstruct 3D information from a single image, the performance is not as good as that of networks that consider several images or take into account the camera motion [22].

Unsupervised learning techniques have been recently proposed, such as a network composed of depth and pose networks with a loss function based on warping views to a target [23]; or another based on generative adversarial networks [24]. Still, unsupervised approaches are not as accurate as recent supervised methods for monocular depth estimation such as BTS [25], VNL [26], DeepV2D [27], or so-called self-supervised methods [28,29,30].

All these deep learning techniques depend on a undetermined scale factor that converts the generated depth maps into absolute values. This is not practical for most cases in robotics since an absolute depth map of the surrounding environment is needed. Pinard et al. recently pointed out this issue [31], solving the problem by adding the velocity of the camera as an additional input. Although the concept is similar to the one proposed in this paper, it should be noted that both the dataset and the objective in [31] are different, since the objective is the estimation of the depth image from the point of view of a drone moving at a constant speed. Both the magnitudes of the inputs and their shape are not applicable to our environment. On the other hand, the distances that are handled in [31] and other related approaches are very different from the working range that we consider since they are too large and coarse. The reason is that their focus is on localisation tasks, while we are dealing with a maximum distance that is determined by the working area of the robot for manipulation within the competition’s deep shelves as apposed to nearly a bird’s eye view in existing datasets. Our approach also solves the above-mentioned problem and absolute distances are provided.

## 2. Methodology

In order to evaluate the importance of considering prior and external models in the design of deep network architectures, we propose three architectures to solve the depth estimation problem in a robot with an eye-in-hand camera for manipulation in an online shopping warehouse shelf (Figure 1). In this scenario, fixed RGB-D cameras or laser sensors have been commonly used to get a faithful 3D representation of its surrounding space in order to deal with a large number of different items [32]. However, those sensors—either fixed or mounted on the robot—suffer from visibility issues to perceive objects such as those occluded or not visible within the shelf. Our overall goal is to propose a different complementary approach by means of an eye-in-hand RGB sensor (Figure 1b) and actively building a 3D representation by moving this visual sensor towards the regions of interest.

Using techniques of data augmentation, a dataset for training was generated. The proposed deep networks are trained and tested with this dataset. As a measure to evaluate to what extent the inclusion of prior knowledge and sensor models in the architectures improves the training performance and the accuracy of the results, we will use a comparison of the final error in the resulting estimation of depth.

### 2.1. Network Architectures

There are previous attempts to estimate a depth image from a single camera using SLAM (simultaneous localisation an mapping) techniques [33,34] or considering bio-inspired models [13]. They are based on the use of camera models and the relationship between the image features and the physical displacement of the camera. These models are integrated in the proposed deep architectures.

**Figure 2:**
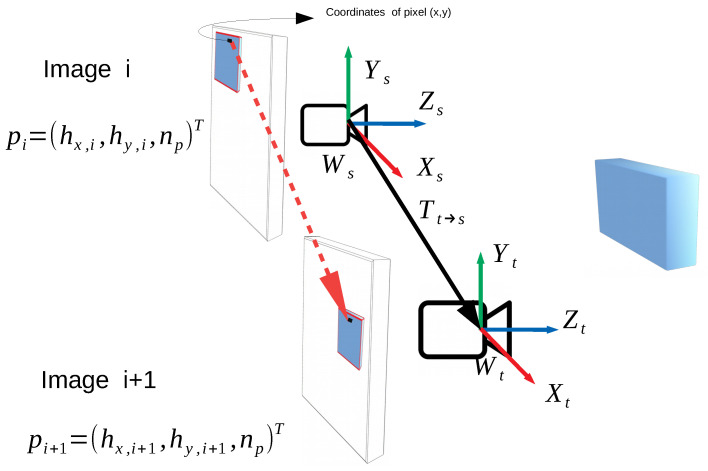
An object is projected onto the image plane of the camera in two consecutive instants (i, i + 1) after a displacement ($T_{s\to t}$) taking as reference the frame source (s) of the camera ($W_{s}$) and considering the target (t) frame of the camera ($W_{t}$), the displacement of the pixel ($p_{i}$) in homogeneous coordinates $h_{x}$, $h_{y}$ and $n_{p}$ (near camera plane), is determined by the difference between the position of the pixel in the images (i + 1) and (i), i.e., $p_{i+1}-p_{i}=\left(T_{t\to s}p_{i}\right)-p_{i}$. *x* and *y* denote the coordinates of the pixel in the image plane.

Our approach builds a 3D representation of the surroundings using a monocular camera mounted on the robot’s hand, differently from approaches using fixed sensors such as RGB-D cameras. Figure 2 depicts the general view of the proposed approach. While moving the robot’s hand towards a target object, first the mounted camera captures a scene of the surroundings (a source image). Then, the hand moves slightly, and the camera captures a new scene (a target image). Simultaneously, a relative pose between those images ($T_{t\to s}$) is calculated based on the joint angles measured by the encoders embedded in the robot’s arm. We convert the relative pose into a displacement map of each pixel in the image plane so that it has the same dimensions as the images. Let us denote by $p_{i}$ and $p_{i+1}$, the homogeneous coordinates of a pixel in the images *i* and *i + 1*, respectively (see Figure 2). Using a transformation matrix from a target view (t) to a source view (s) ($T_{t\to s}$), the displacement of each pixel is defined as follows:

(1) $$Displacement\left(x,y\right)=\left[\begin{array}{c} \Delta px\\ \Delta py\\ \Delta pz \end{array}\right]=p_{i+1}-p_{i}=\left(T_{t\to s}p_{i}\right)-p_{i}$$

To compute this displacement, the model of the camera should be defined. Previous deep learning approaches do not consider the camera model; however, this can be easily obtained from the camera calibration. In our case, we use the the pinhole camera model [35]. The kinematic model should be known to estimate the pose of the camera from the joint values. This is easily derived from the Baxter robot’s kinematics [36]. Of course, this restriction limits the application to this particular type of robot, but the obtained depth map will be valid for real and measurable space.

### 2.2. DepthS Neural Network

The first network design combines the knowledge provided by the camera model with the obtained visual information. The inputs to the network are two consecutive images and the change in the position of each pixel as estimated using the robot and camera models [35,36]. This displacement has three components (${\left[\Delta px,\Delta py,\Delta pz\right]}^{T}$) so that the dimensions of the displacement map are three channels × height × width, as an RGB image. The output is the depth image scaled in a depth range defined by the workspace volume.

**Figure 3:**
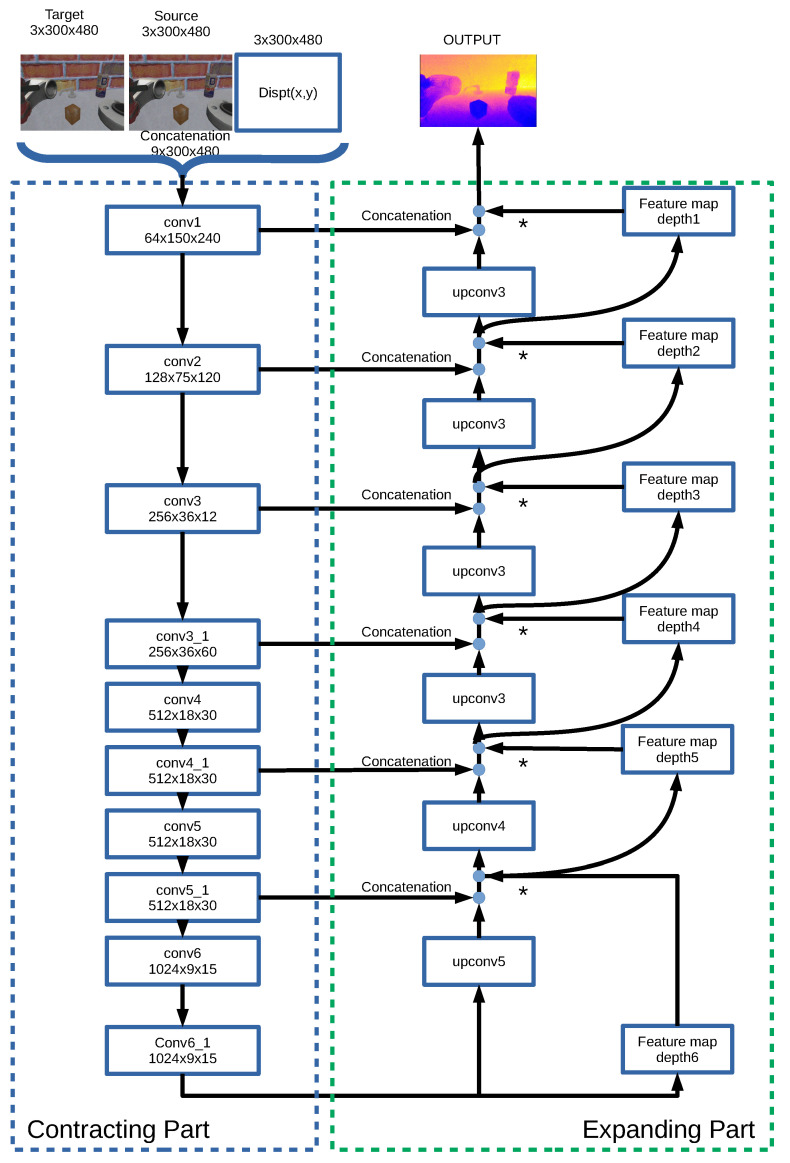
Detailed architecture of depthS. The three inputs (source image, target image and displacement map) are convoluted to extract the local features, and then the features are deconvoluted to generate the depth image. Each box represents a layer of the network where convolutional operations take place in the contractive part, and an unconvolutional and unpooling operations are performed in the expanding part.

The architecture of this network is based on a simple convolutional neural network (CNN). To combine multiple inputs in CNN, a simple choice is to stack all inputs together and feed the CNN with them. This network is illustrated in Figure 3 and we call this architecture *depthS*. DepthS is based on *FlowNetSimple* [37], an approach that fits well with our needs, based on our previous experience. First, depthS concatenates these inputs and convolves them three times before the contracting part. The contracting part is composed of multiple convolutional layers to abstract feature maps. After the contracting part, the feature maps are extended in the expanding part to generate a final depth map. The expanding part is mainly composed of up-convolutions, consisting of unpooling and convolutional layers (Figure 3). To refine the predicted depths, we concatenate the deconvoluted feature maps with two corresponding feature maps: the feature maps from the contracting part of the network, and an upsampled coarser depth prediction. For instance, in Figure 3, the output of *upconv3* is concatenated with the products of *conv3_1* and *depth4*. We repeat this process 5 times. This method of deconvolution was previously used by other researchers [21,22,38]. The contracting and expanding parts are similar to those in [37], though we had to adjust them since the number of inputs is different.

### 2.3. DepthC Neural Network

**Figure 4:**
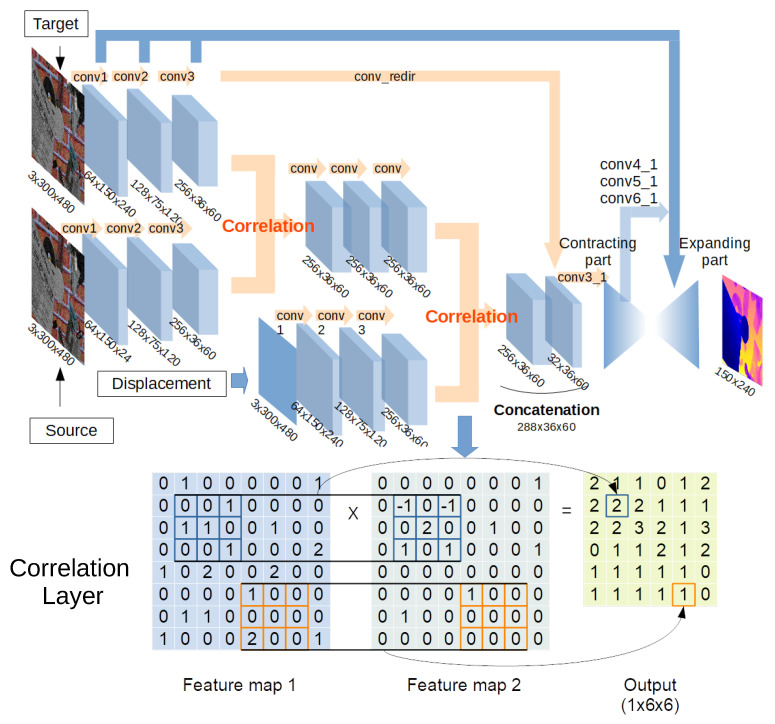
Architecture of depthC. The features extracted from the images are processed by two convolutional branches that are merged by a correlation layer. The result is convoluted and correlated with the third stream of features extracted from the displacement map. An example illustrating how the correlation layer operates is shown at the bottom.

One of our previous models to compute a depth map [13] is included in the design of our second architecture, called *depthC*. In that work, the optical flow and the displacement of the camera are used to generate the depth image for a static scenario. Our approach to estimate the optical flow from two images follows *FlowNetCorr* [37]. Then, this estimation is combined with the displacement input as described above for depthS. As in *FlowNetCorr*, we use so-called *correlation layers*, which perform multiplicative patch comparisons between two feature maps. The overall architecture of *depthC* is shown in Figure 4.

First, depthC processes three inputs with identical streams, a pair of images and a displacement map. Then, feature maps of the images are combined by the first correlation layer. Subsequently, the product of the first correlation layer is convolved three times, and it is associated with a feature map of the displacement at the second correlation layer. Moreover, the product of the second correlation layer is concatenated with a feature map of a target image denoted by *conv_redir* in Figure 4. In this way, the feature map generated from the source image is combined with the features generated from the correlation between the visual displacement and the target image. Finally, contracting and expanding parts process the product and generate a final depth map as in DepthS.

The correlation layer is used to associate two feature maps patch by patch [37]. It convolves a feature map with another map similarly to one step of convolution in a CNN, with the important difference that a CNN convolves inputs with weights, whereas there are no weights in the correlation layer.

### 2.4. DepthCSx Neural Network

**Figure 5:**
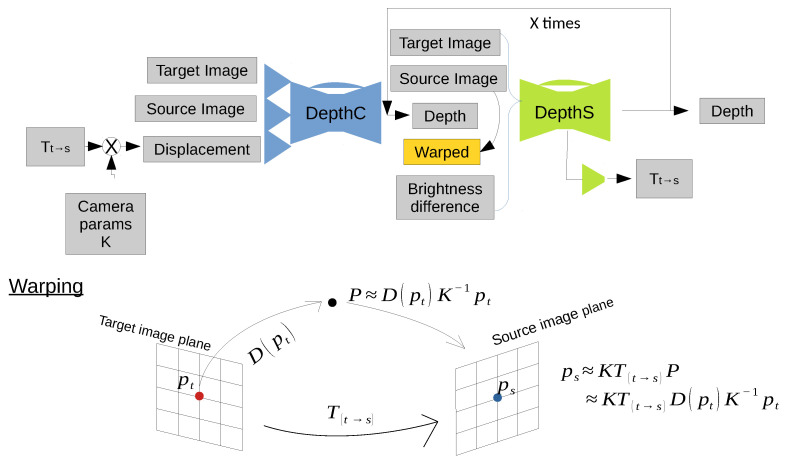
Architecture of depthCSx. DepthC predicts a first depth map, then depthS processes a pair of images, the predicted depth map, a warped image and brightness differences to generate an improved depth map and a new relative pose $T_{t\to s}$. We iterate depthS x times so that depth maps and relative poses are improved repeatedly.

This network combines a single depthC with multiple depthS networks. Its design was inspired by FlowNet2 [39], DeMoN [22] and SfM-Learning [23]. This type of network is denoted by depthCSx, where x is the number of depthS networks used in the network design. The architecture of depthCSx is shown in Figure 5. To combine two different neural networks effectively, we introduce a brightness error and a subconvolutional neural network diverging from the mid of depthS, which computes a relative pose. First, depthC processes a pair of images and a displacement map, and predicts a first depth map. After obtaining the first depth map from depthC, depthS processes the following information: a pair of images, a predicted depth map, a warped image and brightness differences. To effectively link the first depth map with a pair of images, we introduce a warped image and a brightness error [23,39]. The warped image ${\tilde{I}}_{w}$ is obtained from a target view $I_{t}$ by projecting pixels onto the source view $I_{s}$, based on the predicted depth map $\hat{D}$ and relative pose ${\hat{T}}_{t\to s}$ and using bilinear interpolation to obtain the value of the warped image ${\tilde{I}}_{w}$ at location $p_{t}$. To do so, we define $p_{t}$ as the homogeneous coordinates of a pixel in the target view, and *K* is the camera intrinsic matrix. The coordinates of captured objects in the target view *P* are expressed with the projected coordinates of $p_{t}$, the predicted depth of the object $\hat{D}\left(p_{t}\right)$ and the camera matrix *K* as follows:

(2) $$P∼\hat{D}\left(p_{t}\right)K^{-1}p_{t}.,$$

where *P* is calculated on the target view’s camera frame coordinates. Then, we can calculate the projected coordinates of $p_{t}$ onto a source view $p_{s}$ as follows:

(3) $$\begin{array}{cc} p_{s} & ∼K{\hat{T}}_{t\to s}P∼K{\hat{T}}_{t\to s}\hat{D}\left(p_{t}\right)K^{-1}p_{t}, \end{array}$$

(4) $$\begin{array}{cc} {\tilde{I}}_{w} & =\sum_{i\in top,bottom,j\in left,right}w^{ij}I_{s}\left(p_{s}^{ij}\right), \end{array}$$

where $w^{ij}$ is the approximate value between projected and neighbouring pixels, which sums up to 1; *i* and *j* refer to relative pixels around the current pixel (x,y) and in the sum *i* and *j* vary from top to bottom and from left to right, respectively, since they are references relative to pixel (x,y). The brightness error $L_{bright}$ is an absolute mean of differences between $I_{t}$ and ${\tilde{I}}_{w}$ on each pixel, expressed as follows:

(5) $$L_{bright}=\frac{1}{wh}\sum_{x,y}\left|I_{t}\left(x,y\right)-{\tilde{I}}_{w}\left(x,y\right)\right|,$$

where *w* and *h* are the width and height of an image, respectively, and *x* and *y* are the coordinates of each pixel in the image.

Thus, depthS processes a pair of images, a predicted depth map, a warped image, and brightness differences together, and it generates an improved depth map and a new relative pose $T_{t\to s}$. The new $T_{t\to s}$ is calculated from the subconvolutional network diverging from the mid of depthS. We iterate depthS multiple times so that depth maps and relative poses are improved repeatedly. The updated relative pose and predicted depth map are used to compute a new warped image and brightness difference with Equations (3) and (5). Next, depthS processes the new warped image and brightness differences along with a pair of images and previously predicted depth maps repeatedly.

## 3. Experimental Setup

The considered scenario is a robot arm with an eye-in-hand camera for manipulation in a confined space. Under these conditions, the goal is to estimate a dense and faithful depth map, especially in the space within reach of the robot’s gripper since this is critical for succefully grasping a target object.

### 3.1. Dataset Generation

Most existing datasets for monocular depth estimation were designed for the localisation of vehicles or mobile robots. The distances in the dataset samples are too large and coarse for our manipulation application. This is the case for the most popular datasets for monocular depth estimation, such as the KITTI [8] or the Pandora [9] datasets. It is clear that these datasets cannot be used to train the network architectures that we propose, in which the images are captured by a video camera on a robotic arm that produces small displacements. Therefore, we need to generate our own small, ad-hoc dataset. To do so, we used the Gazebo/ROS simulator [40] with a Rethink Robotics Baxter with which it is ideally possible to get the ground truth for depth maps, camera poses and images. A model of Baxter allows to transform from joint space to camera poses. The environment in front of the robot is limited by a wall and two types of walls were used in the dataset generation.

**Figure 6:**
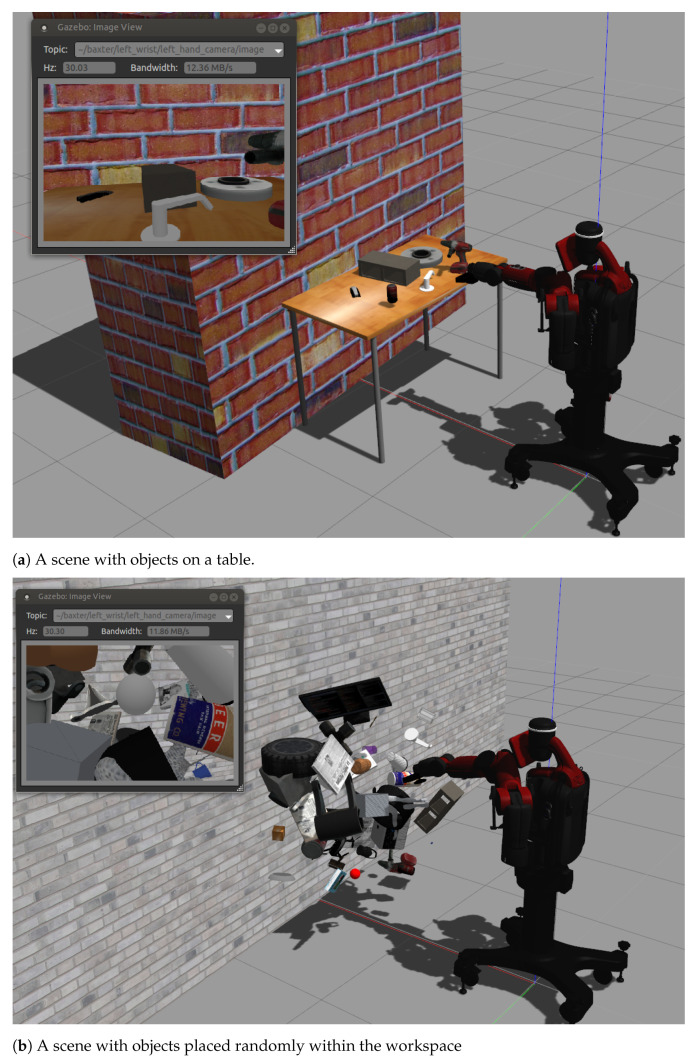
Examples of generated scenes for the dataset. The insets show the images captured by the eye-in-hand camera.

In order to have enough diversity in the dataset, 30 ordinary object models were used such as a newspaper, book, bowl, cinder block, cube, ball, pencil, etc. In our first trials, the objects where placed on a table (Figure 6a), randomly changing their poses as well as the objects present in each scenario. Subsequently, the number of samples in the training dataset was increased with data augmentation techniques. In particular, the objects were placed randomly in front of the robot within the workspace volume (Figure 6b) so that their depths spread over the robot visual area.

A depth camera simulation placed in the location of the RGB camera was used to capture the depth images and generate the ground truth. The movements of the arm are limited in such a way that an initial pose is defined to capture the maximum area of the workspace, and then different poses are generated into a sphere centerer in the initial pose and with maximum radius of 5 cm. The selection of this value was based on geometric calculations from camera angular FOV, the limitations of Baxter, and the nature and size of the workspace defined by the shelf. The 6 DoF of the camera are randomly changed within the limits of this sphere, in such a way that the end effector is moved within these limits while keeping the orientation of the camera unchanged, so that only the translational component will need to be input into the neural networks, reducing their complexity. This point differs significantly from the case proposed in [31] in which the camera is mounted on the drone and its speed is used for distance estimation. In addition, the total displacement values there are up to 30 cm.

To reproduce the noise present in a real setting, Gaussian noise was added to images and relative camera poses. The standard deviation used to generate the white noise was 0.07 for the images and 1 mm for the poses. The values of these deviations are referred to the official hardware description of Baxter. The procedure to generate the dataset is divided into three steps:

1. The robot captures the first image with the monocular camera embedded in its arm.
2. The camera is moved slightly and captures the second image with its depth map. The relative camera pose is saved as well. This pair of images, the depth map for the second image, and the relative pose are stored as one element in the set of data.
3. A new scene is generated by randomly shuffling objects.

Finally, our resulting dataset is composed of 10,000 such elements (for 5000 scenes with objects on a table and 5000 with objects in the workspace), which is one or two orders of magnitude smaller than the typical datasets for deep learning. For example, the KITTI dataset [8] for depth estimation and prediction contains 93,000 depth images and the Pandora dataset [9] is composed of 250,000 images; both of them are oriented to depth estimation by moving vehicles. We split the dataset into 8000 and 2000 sets for training and validation, respectively. In addition, we prepared 600 additional samples for inference.

### 3.2. Training Setup

#### 3.2.1. Loss Functions

The root mean square error (RMSE) of depth is adopted as loss function because it is one of the most commonly used and, therefore, it is convenient for comparisons. Depth RMSE is calculated using a predicted depth $\hat{d}$ and ground truth *d* as follows:

(6) $$L_{rmse}=\sqrt{\frac{1}{wh}\sum_{i=1}^{w}\sum_{j=1}^{h}{\left({\hat{d}}_{i,j}-d_{i,j}\right)}^{2}},$$

where *w* and *h* are the width and height of a depth map, respectively.

In addition to RMSE, the smoothness error is used. This can be considered as L1 norm of the second-order gradients for the predicted depth maps [23,38].

For the training of depthC and depthS, we used only depth RMSE, whereas smoothness loss and brightness loss are used in the training of depthCSx. Hence, the loss function for an iteration *i* of depthCSx is the weighted sum of loss functions expressed as follows:

(7) $$L_{final}^{i}=w_{rmse}^{i}L_{rmse}^{i}+w_{bright}^{i}L_{bright}^{i}+w_{smooth}^{i}L_{smooth}^{i},$$

where $w^{i}$ are weights for each loss function, and the superindex *i* refers to the current depthS iteration in depthCSx network, as explained in Section 2.4 and shown in Figure 5.

#### 3.2.2. Optimizer and Regularization

To circumvent overfitting and learn a depth map efficiently, the Adam optimizer [41] and regularization techniques were applied. A stochastic gradient descent method (SGD) was first tested but the Adam optimizer converged faster.

As for regularization in the training process, two techniques were used: L2 regularization gives a penalty on a loss function with the coefficient of a sum of squared weights. The selected value for L2 regularization used for training was $10^{-4}$. The second technique was dropout [42]. Moreover, a normalisation technique was also used. In particular, group normalisation [43]. The size of the group was configured as 16. As usual, the choice of these two values was based on a prior systematic tuning of the parameters of the neural network.

### 3.3. Evaluation Metrics

To analyse the results with multiple criteria, several evaluation metrics were computed: L1-inv, L1-rel, sc-inv amd RMSE [22,23,44]. L1-rel calculates a depth error relative to the ground truth. L1-inv can relatively increase if there is a large error for small values of depth. Finally sc-inv is a scale-invariant error introduced [33].

### 3.4. Training and Validation

DepthS, depthC, depthCS and depthCSS were trained with the generated dataset (the code is available in: https://gitlab.com/aduranbosch/depthCSSx (accessed on 19 February 2021)). The above evaluation metrics were used to analyse the results from multiple viewpoints. The results are separated into training and validation phases as well as an inference phase. In one epoch of training and validation, the networks were first trained on the 8000 scenarios in the training set, and the learning was evaluated on the 2000 scenarios in the validation set. The best weights in the validation were saved for inference. For the inference, the networks tried to predict depth maps on additional 600 unseen scenarios.

#### 3.4.1. DepthC and DepthS

First, we trained depthS and depthC to evaluate how much the correlation layers contribute to the performance. Both networks were trained for 29 epochs with the dataset. The parameters used for training are summarised in Table 1.

#### 3.4.2. DepthCSx

Next, we trained depthCSx. To do so, first depthCS (one depthC + one depthS) was trained for 30 epochs, reusing the best weights learned previously for depthC. In the training of depthCS, we only optimised the weights of depthS while fixing the weights of depthC. After the training of depthCS, we moved to training depthCSS, which stacks one depthC and two depthS. We similarly optimised only the second depthS for 30 epochs, while using the best weights of depthCS learned previously. The parameters of this training are shown in Table 1

## 4. Results

### 4.1. Training Progress

#### 4.1.1. DepthS and DepthC

**Figure 7:**
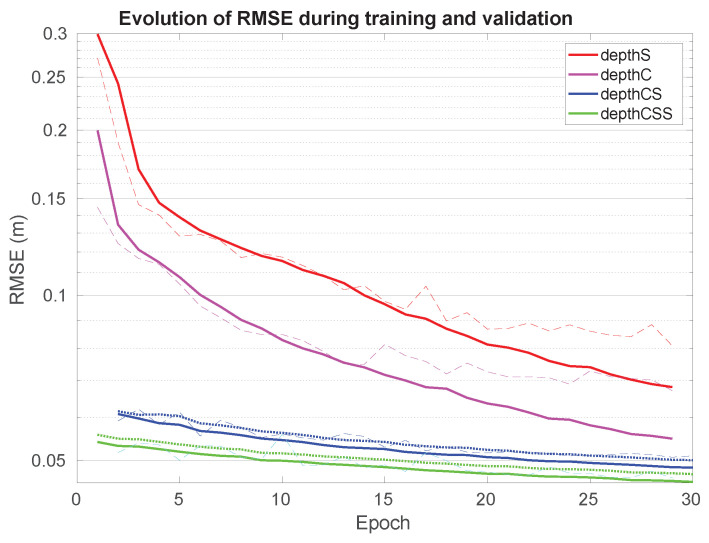
Evolution of RMSE for the proposed neural networks during training and validation. Dashed lines correspond to validation, solid lines to training, and dotted lines represent the sum of the weighted loss functions.

From Figure 7, it is apparent that depthC outperforms depthS in both training and validation scores. Also, it can be observed that the training and validation scores converge with a very small oscillation. However, the validation scores did not decrease well after around 15 epochs.

The training progress along 30 epochs for depthCS and depthCSS is shown in Figure 7 (blue and green curves) both for the sum of loss functions (dotted lines) and RMSE (solid lines).

#### 4.1.2. Sensitivity Analysis to Dataset Size

**Figure 8:**
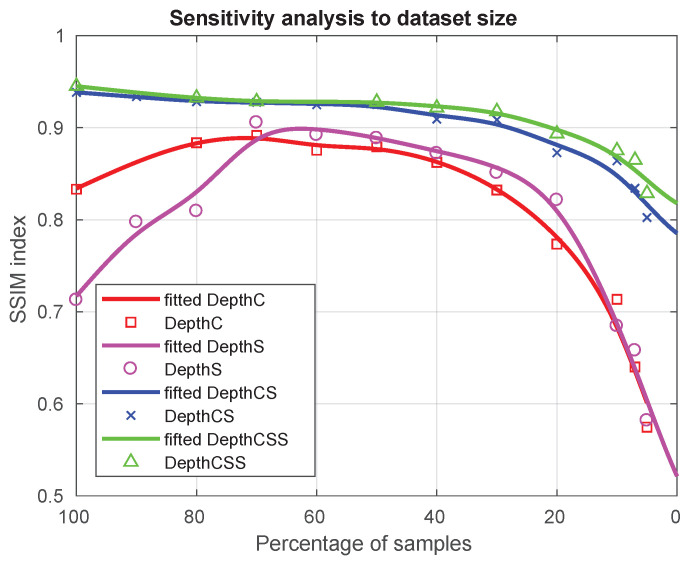
Sensitivity analysis to dataset size. The plots show the estimation of the SSIM index for each neural architecture versus the percentage of samples (number of scenes in the training and validation datasets) using as reference the maximum size of 10k. Note how with only 3000 samples (30%) the SSIM index is already over 0.9 for depthCS and depthCSS and it keeps over 0.8 for only 1000 samples (10%). Even for DepthC and DepthS, the SSIM index is close to 0.9 for 7000 samples (70%), though it decreases for larger sizes, most probably due to overfitting.

We trained the proposed networks using different fractions of the main dataset of 10,000 samples. We then evaluated their performance using a test set that had not been used for any of the training sessions and that, although it is similar in the typology of objects, they are not the same as those used in the training parts. The structural similarity index (SSIM) is used to assess the results [45]. SSIM provides us with information about the structural similarity between the depth image generated by the neural networks and the ground truth. The main advantage of this index is that its range of possible values extends from 0 to 1, and two images are more similar the closer SSIM is to 1. The plots of the percentages with respect to the maximum size of the dataset versus the estimation of the SSIM index for each neural architecture are shown in Figure 8.

### 4.2. Evaluation Metrics

**Figure 9:**
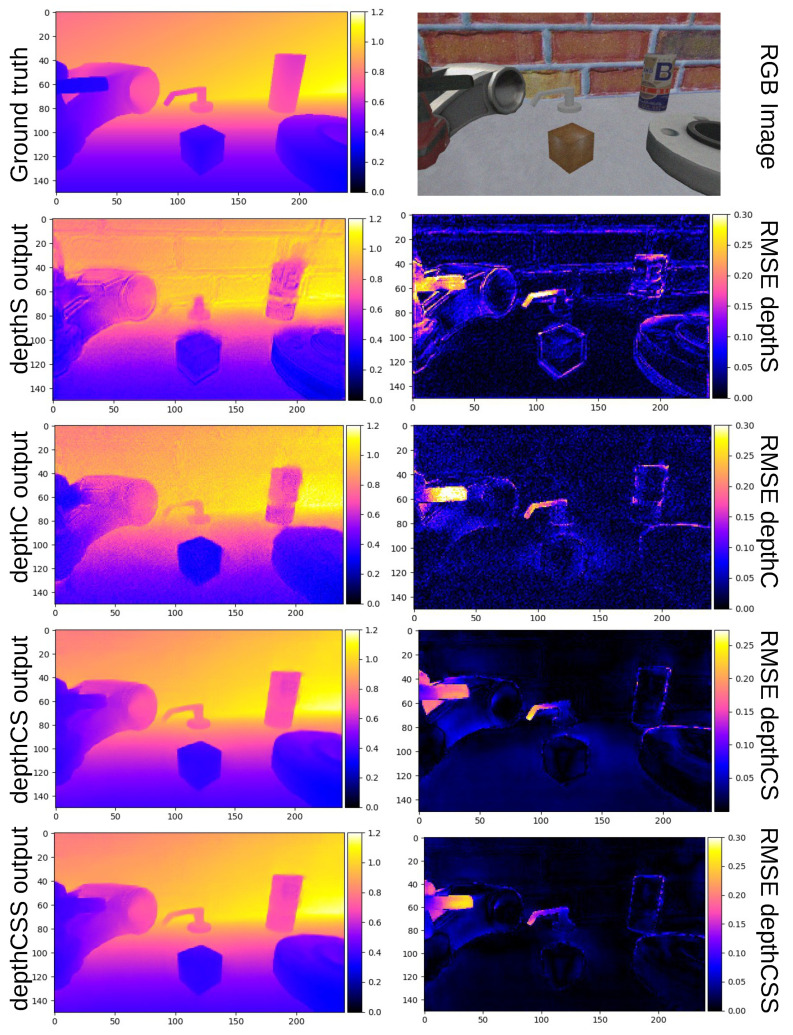
Representative example of depth maps predicted by depthC, depthS, depthCS and depthCSS. The ground truth and RGB image are also shown. The units of the color bars on the right are meters. Closer distance is colored in blue and farther distance in red and yellow. The RMSE value for each pixel is shown in the images in the right column.

After training the different deep networks, the established metrics are used for estimating the inference error of the testing dataset. These results are shown in Table 2. One representative example of the depth maps predicted by DepthS, DepthC, DepthCS and DepthCSS is displayed in Figure 9 along with the ground truth and the target image. The target image was captured by the monocular camera at a given moment. The ground truth was provided by a simulated depth camera located at the same coordinates as the eye-in-hand camera. The depth maps are colored to visualise the distance from the camera.

## 5. Discussion

Beyond the interest of the proposed approach for our particular application domain, the results in Table 2 support the hypothesis that integrating prior knowledge and sensor models relative to robotic active vision into deep learning can significantly improve performance. The three neural networks have been trained with the same number of epochs.

Also, leveraging the camera model in depthS, allows us to define the absolute units of the obtained depth images (Figure 3); a clear advantage over alternative deep learning methods, for which a scale factor is needed to estimate the real distance. For depthS and depthC, the training parameters and procedures are similar. However, the incorporation in the depthC design of the model of depth estimation from optical flow and the displacements of the camera improves its performance with respect to depthS around 25% (as measured with RMSE). Embedding prior knowlegde in depthC from two previously established rules for the estimation of optical flow from two images and the correlation of this magnitude with the variation of the camera position allowed us to define the correlation rules between these elements. The correlation layers extract features of each input before the layer and link them patch by patch in the layer. On the other hand, depthS failed to properly learned to link these features through training by itself. This result suggests that our approach contributes to a better accuracy and the reduction of training time.

Also, depthCSx is a combination of the two previous architectures, but with the incorporation of the roto-translation camera model to estimate the change of position of each pixel of the first image, using the depth prediction provided by depthS. By itself, this fact implies the use of prior knowledge represented by the pre-trained depthS, and an additional source for the improvement of performance. The way the camera model predicts the pixel variation from the depth estimated by depthS, is crucial to enhance the global network performance: around 10% with respect to depthC and 34% over depthS, in terms of RMSE.

From these results, it can be drawn that as models such as the camera model (depthS), the estimation of optical flow and variation of the position (depthC), and the model of the estimation of the translation of the camera from a depth image, are incorporated in the network designs, the resulting performances are better for the same dataset.

Though it is customary to asses the performance of a system by means of comparisons with other methods, this endeavour turned out to be impossible in our case, and our preliminary tests using some methods proposed in the literature did not generate reasonable results. The main reason is that those methods are based on training the network with images from video sequences generated for a constant camera displacement over the scenario, whereas in our case the camera executes random oscillatory movements around a point and, therefore, our dataset is not compatible with them. Comparing directly our RMSE values with those reported by state-of-the-art methods—such as Pinard et al. [31], BTS [25], VNL [26], or DeepV2D [27]—would also made no sense—even though our values are much smaller—since it would simply mean comparing two problems that do not have the same scale. Similarly, using standard datasets—such as KITTI [8] or Pandora [9]—for the sake of comparison is not an option either, since they do not match the requirements of our use-case in terms of the work environment or necessary camera displacements, as already mentioned.

Finally, our results have been obtained with an ad-hoc dataset that is one or two orders of magnitude smaller than the typical datasets used in deep learning. This fact suggests that the incorporation of prior knowledge and sensor models relative to robotic active vision can contribute to the development of sample-efficient deep learning by significantly reducing the size of the required datasets. The results of our sensitivity analysis to dataset size clearly confirm this claim, since with only 3000 samples the SSIM index is already over 0.9 for depthCS and depthCSS and it keeps over 0.8 for only 1000 samples (Figure 8). This promising results suggest the feasibility of physically generating the ad-hoc data in the real world in the near feature, and resorting to data augmentation when necessary.

## Figures and Tables

**Table 1:** Training parameters.

Parameter	DepthS	DepthC	DepthCSx
Regularization			
L2	$10^{-4}$	$10^{-4}$	$10^{-4}$
dropout	0.0	0.0	0.5
Adam			
learning rate	$10^{-3}$	$10^{-3}$	$10^{-3}e^{\left(-0.95*ep\right)}$
Beta	(0.9,0.999)	(0.9,0.999)	(0.9,0.999)
Coefficient of loss			
$w_{rmse}$	1.0	1.0	1.0
$w_{bright}$	0.0	0.0	0.01
$w_{smooth}$	0.0	0.0	0.01

**Table 2:** Results for error metrics.

Network	Error Metrics			
	RMSE (m)	L1-rel	L1-inv	SC-inv
depthS	0.1173	0.1650	3.1450	0.1767
depthC	0.0856	0.1219	0.9603	0.1240
depthCS	0.0766	0.1089	0.7879	0.1183
depthCSS	**0.0732**	**0.1050**	**0.7649**	**0.1119**
